# A Comprehensive and Universal Method for Assessing the Performance of Differential Gene Expression Analyses

**DOI:** 10.1371/journal.pone.0012657

**Published:** 2010-09-09

**Authors:** Mikhail G. Dozmorov, Joel M. Guthridge, Robert E. Hurst, Igor M. Dozmorov

**Affiliations:** 1 Department of Arthritis and Immunology, Oklahoma Medical Research Foundation, Oklahoma City, Oklahoma, United States of America; 2 Department of Urology, Oklahoma University Health Sciences Center, Oklahoma City, Oklahoma, United States of America; 3 Department of Biochemistry and Molecular Biology, Oklahoma University Health Sciences Center, Oklahoma City, Oklahoma, United States of America; Victor Chang Cardiac Research Institute, Australia

## Abstract

The number of methods for pre-processing and analysis of gene expression data continues to increase, often making it difficult to select the most appropriate approach. We present a simple procedure for comparative estimation of a variety of methods for microarray data pre-processing and analysis. Our approach is based on the use of real microarray data in which controlled fold changes are introduced into 20% of the data to provide a metric for comparison with the unmodified data. The data modifications can be easily applied to raw data measured with any technological platform and retains all the complex structures and statistical characteristics of the real-world data. The power of the method is illustrated by its application to the quantitative comparison of different methods of normalization and analysis of microarray data. Our results demonstrate that the method of controlled modifications of real experimental data provides a simple tool for assessing the performance of data preprocessing and analysis methods.

## Introduction

The number of methods available for pre-processing and analysis of high-dimensional data continues to increase, making a comparative assessment of the performance of these various methods increasingly important. Such a comparison should be qualitative, as well as time-, computation- and cost effective. Currently, no commonly accepted rules exist for such comparative testing. The performance of each test should be characterized in terms of the test's ability to distinguish true changes from noise that appears to represent a pattern. The receiver operating characteristic (ROC) curve, which represents all possible combinations of the relative frequencies of the various kinds of correct and incorrect decisions, is usually employed as a simple empirical description of these characteristics [1], [2], [3]. The ROC-curve presents the relationship between sensitivity and specificity and can be used to characterize the overall performance of different arrays and the software designed for data acquisition on the different platforms. However, the lack of simple inferential procedures to discriminate between true and false selections has limited the practical utility of ROC curve analysis. Generation of the decision rules used to estimate the proportions of true and false selections requires knowledge of the distribution of these categorical assessments, which is not provided by the tests themselves. Neither visual inspection of the array images [4] nor the use of differences between perfect matches and mismatches on Affymetrix arrays provides an objective measurement to make these discriminations.

One promising approach is based on the use of a simulation strategy that constructs more-or-less realistic data models with varying statistical characteristics that reflects the properties of real gene expression data [5], [6], [7], [8], [9]. However, such approaches are not fully satisfactory because they rely on model assumptions that are not necessarily supported by empirical studies. The true changes in expression are not known beforehand and largely differ between each experimental situation. Thus, these changes in expression cannot be characterized and used for evaluation *a priori*, and such comparison approaches do not guarantee complete similarity between the structure of the simulated data and complex real-world expression data. The most objective discrimination between false and true changes in a dataset was achieved by using a “spike-in” experimental procedure based on Affimetrix GeneChip technology [10], [11], [12]. This approach generated objectively different signals by changing the mRNA concentration in a controlled quantitative manner. Data from spike-in experiments (where the mRNA-ratios of a set of artificial clones are known) can be used to determine the relative merits of a set of analysis methods [13], [14]. The design of a spike-in experiment must be based on assumptions as to how real microarray data behave. These assumptions are generally less restrictive than those required to simulate microarray data. However, this spike-in approach remains expensive, time consuming and inflexible, thus limiting its utility.

We propose a simple approach to assessing various methods of data analysis that is based on the use of real microarray data. Our assessment can be easily implemented into the design of any microarray experiment and can be performed with minimal training. It is important to note that our approach is applicable to any microarray dataset generated from homogenous groups of samples hybridized to the arrays.

The first step in our strategy is the introduction of controlled changes into homogeneous group of microarray data. The group is split into two equal subgroups, one of which remains unchanged and is used as a standard for comparison. The second subgroup is altered by introducing controlled fold changes in the gene expression values. Similar step - introduced changes in the real gene expression data - was used earlier for demonstration of the breakdown of Lowess normalization after “one direction changes” [15]. Our implementation introduces these controlled fold changes homogenously over the entire range of gene expression levels. This modification was applied to 5–20% of the entire dataset, thereby retaining the complex structure and statistical characteristics of the remainder of the data.

We illustrate the application of this method for an optimal cutoff estimation, intensity-based filtering and for optimal fold differences selection that provides the highest ratio of true to false signals. This method enables us to estimate the degree to which the quality of the analysis depends on the level of gene expression. Our method estimates the minimal necessary number of replicates to achieve an expected sensitivity in the differential gene expression analysis. We also applied our approach to the comparative estimation of the quality of data preprocessing (normalization) and the performance of the methods of differential gene expression analysis.

## Materials and Methods

### Global gene expression profiling

We performed gene expression profiling from 20 samples of Epstein-Barr Virus-transformed B cells collected from normal healthy donors. RNA was isolated using Ambion's RNAqueous RNA isolation kit (Ambion Inc., TX) according to the manufacturer's protocol. After purification, RNA concentration was determined with a Nanodrop scanning spectrophotometer. Illumina Whole Genome Human Ref-8 v2.0 arrays containing ∼24,000 probes were handled according to standard protocols established by the manufacturer. Briefly, 200ng of total RNA from each sample were used to generate biotin-labeled cRNA probes using an Illumina MessageAmp cRNA labeling kit protocol (Ambion). Quality control of the cRNA was performed using an Agilent Bioanalyzer and a Nanodrop. Labeled cRNA probes were hybridized to Illumina arrays and images were obtained on an Illumina Beadchip scanner. The images and raw data from each chip were transferred automatically to the microarray database using one of the specified microarray core servers. The raw data is available on GEO [16] (GSE22630 accession number) and in Supplemental Table S1.

### Microarray data analysis

Our methods for data normalization and analysis are based on the use of “internal standards” [17] that characterize some aspects of the system's behavior, such as technical variability, as presented elsewhere [18], [19]. In general, an internal standard is constructed by identifying a large family of genes that behave similarly. Genes expressed below technical sensitivity represent one example of an internal standard. This group of genes comprises a background cohort that conforms to the parameters of normal distribution. Another example is a group of genes with similar expression patterns across several distinct experimental conditions, denoted as an equally expressed cohort. These internal standards are used to robustly estimate parameters that describe some features of the experimental system, such as the pattern of genes expressed distinctly from background, cohort of stably expressed genes, or genes displaying similar dynamic behavior.

#### Two-step normalization procedure

The first step is determination of the parameters of a background of an array – average (Av) and standard deviation (SD) – is performed using a special iteration procedure. Data in each array are transformed to make these parameters equal to 0 and 1, correspondingly. After this transformation gene expression data are presented in the units of standard deviation of the background. We accept the threshold of 3 SD above the mean of background distribution as the preliminary criterion for distinguishing between expressed and non-expressed genes. Only genes expressed above background are used for the second step.

The second step is the adjustment of normalized profiles to each other by robust linear regression. This procedure is based on the selection of equally expressed genes as a homogenous family of genes with normally distributed residuals defined as deviations from the regression line. The parameters of this distribution are obtained by the iterative procedure similar to one used for the selection of normally distributed background noise. Outliers are thereafter determined as having deviations **not associated** with this internal standard of equality of expression.

The differential gene expression analysis – Associative analysis [18], [19] - includes the following steps:

- Construction of the ‘reference group’ by identifying a group of genes expressed above background with inherently low variability as determined by an F-test. The ‘reference group’ presents an internal standard of equal expression. As such, the ‘reference group’ is used to assess the inherent variability resulting from technical factors alone (technological variation). By creating an estimate of the technological variation we are able to select a group of biologically stable genes.
- Selection of replicates using the commonly accepted significance threshold of p<0.05 with a Student T-test. This selection maintains the commonly accepted sensitivity level; however, a significant proportion of genes identified as differentially expressed at this threshold will represent false positive determinations.
- An Associative T-test in which the replicated residuals for each gene from the experimental group are compared with the entire set of residuals from the reference group defined above. The *Ho* hypothesis is checked to determine whether the levels of gene expression in the experimental group presented as replicated residuals (deviations from the averaged control group profile) is associated with a highly representative (several hundred members) normally distributed set of residuals of gene expression values in the reference group. The significance threshold is then corrected to render the appearance of false positive determinations improbable. Only genes that pass both tests are presented in the final selections.

The two-step normalization procedure and the Associative analysis functions are implemented in MatLab (Mathworks, MA) and available from authors upon request. These algorithms are also obtainable from an R package *diffGeneAnalysis*, available as a part of Bioconductor packages (http://www.bioconductor.org/packages/2.5/bioc/html/diffGeneAnalysis.html). Other Bioconductor packages (affy, limma, vsn) were used for Quantile, Lowess and VSN data normalization and for Limma analysis. SAM: Significance Analysis of Microarrays was used as an Excel add-on, downloaded from http://www-stat.stanford.edu/~tibs/SAM/.

### Introduction of balanced changes into the gene expression data

**Figure 1:**
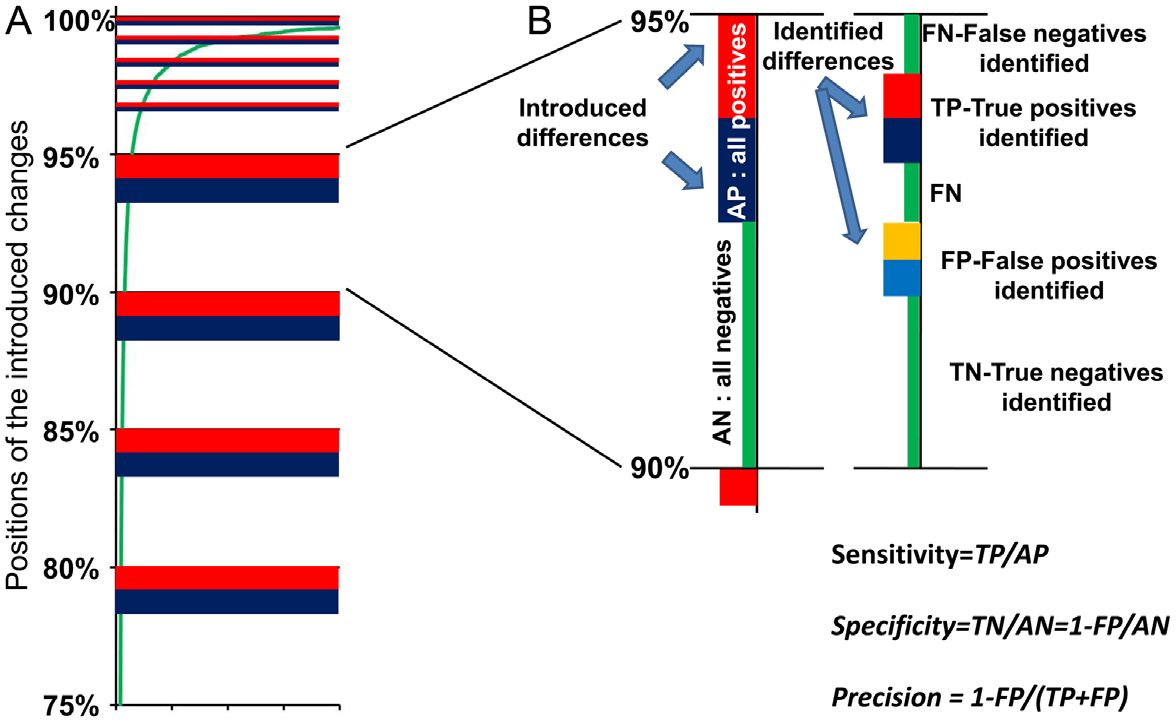
Test system for determination of the Sensitivity and specificity of the differential gene expression analyses. A supposedly homogeneous group of samples was divided into two equal subgroups, one of which was not changed and used as a control and the other used as an experimental group with introduced changes (Supplemental Tables S2, S3, S4 and S5). All data (∼20,000 genes) were divided into 20 equal blocks. A) A fragment of experimental data set. The data are sorted according the averaged level of expression (green line, shown relative to the maximum expression level units). The figures on the left side of vertical axis show the positions of each block (in percents of the total data). Positive (red) and negative (blue) changes were introduced in the 20% portion of genes in the block (usually changes applied to the genes with highest expression in each block, excluding the first one containing the very first 100 genes with highest expression levels. Here 40 differentially expressed genes created within each of five 200 gene segments). B) Structure of one of the blocks (90–95% of data). Left vertical axis presents positions of positive (red) and negative (blue) introduced changes with the rest (green) positions of unchanged gene expressions. Right vertical axis shows selections made by an analysis: red/blue – correct selection of +/− changes, yellow/light blue – false selection of +/− changes among not changed genes, green marked FN – false negative selections (not identified + or − changes), green marked TN – true negative selections. Sensitivity of selections is determined here as a proportion of true positive selections within all produced changes, Specificity determined as a a proportion of true negative selections among all unchanged genes, and Precision is determined as a proportion of true positive selections among all selections made by an analysis, or as a value whose deviation from 1 is associated with the presence of false positive selections.

In this study, we used gene expression data from 20 total RNA samples from Epstein-Barr Virus-transformed B cells collected from normal healthy donors. This presumably homogenous group of samples was sorted by average expression level and split into two equal subgroups, designates as “control” and “experimental”. The data was then split into blocks of 1,000 genes each. Controlled balanced (+/−) changes were introduced into 20% of the data in the experimental subgroup. One hundred genes in each 1000 gene block were modified by a positive change (multiplied by fold change) and another hundred genes were modified by a negative change (divided by fold change). The top thousand genes with the highest expression levels were treated slightly differently: they were split into five 200-gene blocks, and within each of them 20 genes were modified by positive change (multiplied by fold change) and 20 genes were modified by negative change (divided by fold change) (data with color-coded modifications used in this paper are presented in Supplemental Tables S2, S3, S4, S5 and S6). The rationale behind it is that a very wide range of gene expression changes present at the highest expression levels (Figure 1). Therefore, more detailed controlled changes are necessary to better assess performance dependence of an analysis from expression level.

The altered genes are denoted as all positive genes (AP-genes) in contrast to the remaining genes that were initially not changed (all negative – AN-genes). One block of AP/AN genes is shown on Figure 1B. The modification did not noticeably alter the frequency distribution histogram of the data (data not shown), as would be expected from the relatively modest amount of change (20% of genes altered). After applying the analysis procedure, the resulting selections are compared with the AP- and AN-genes for determination of the Sensitivity and Specificity of a given analysis. Sensitivity and Specificity can be expressed using known numbers of true and false positive and negative selections. Here, true positives (TP) are selections made in the course of differential expression analysis among the AP-genes. True negatives (TN) are genes that were not selected as differentially expressed among the AN-genes. False positives (FP) are genes selected in the course of analysis as differentially expressed from the AN-genes. False negatives (FN) are AP-genes not selected as differentially expressed. Given these definitions, we derived the following equations (4):
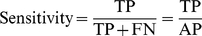


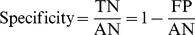



However, the use of Specificity expression as presented here was not particularly useful for characterizing the quality of microarray data analyses. The problem with this metric is that in any microarray analysis, the number of AN-genes always exceeds the number AP-genes by at least several fold. As a result, a significant number of false negative selections will produce only a small deviation from 1 in Specificity value. For example, if there are two hundred differentially expressed genes from twenty thousand total genes measured in an array, selection of ten false positives will result in a Sensitivity of 0.95. In contrast, the same number of false negatives will produce a Specificity value of 0.999. This Specificity value does not express the real impact of this number of selected false negatives, which should be more comparable to the impact of false positive selections. To improve this situation, we used instead the Precision parameter, which is a better characteristic for testing exactness or fidelity in cases with significant differences in sizes of positive and negative numbers. Accordingly [20], Precision is calculated as follows:
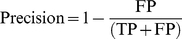
with reciprocal dependence on FP and approaching 1 when FP is close to zero. Now both Sensitivity and Precision are presented symmetrically in the proportions of the true/false positives to all selections.

To estimate stability of the proposed characteristics, our test procedure can be repeated several times with random permutation of the order of samples within the group before each split. The averaged Specificity and Precision parameters from multiple tests, together with their Standard Deviations, are presented in the figures.

## Results

### Influence of fold change and expression level restrictions on the quality of analysis

The data that include controlled balanced changes in the expression levels of a small proportion of the genes in the array can be used for a variety of purposes, including comparison of different methods of data pre-processing and analysis. We begin, however, with a demonstration of the potential of this method for estimation of the effects of different restrictions frequently used in differential gene expression analyses.

The step typically following completion of a differential gene expression analysis using strong statistical criteria is to focus on the most prominent changes. To achieve this some additional restrictions are applied, such as minimal level of expression and the fold changes in the level of expression deemed biologically significant. The influence of these pre-processing steps on the quality of analysis has not been extensively studied.

Filtering by a minimum expression level obscures the influence of extreme variability of low expressed genes. Additionally, filtering out genes that are expressed only at low levels unambiguously demonstrates that the most important biological changes are usually well represented by genes with high expression level. However, the cost of this restriction is that changes in regulatory genes expressed at low levels, such as transcription factors [21], [22], may be lost.

Statistical analysis of differences in gene expression can identify even minimal changes in levels of gene expression if those changes are extremely stable across replicated experiments. However, the statistical significance of a fold change does not necessarily reflect biological relevance. Genes with low fold change differences should certainly be excluded, at least at the stage of initial examination of the results. Those genes displaying high fold changes in gene expression (and, hence, in mRNA abundance) potentially represent the most important functions in the biological system. We refer to these as “beacon genes” [23]. At the same time, changes in the expression/activity of regulatory genes, which are usually neither highly expressed nor display prominent variations in expression levels, may represent important biological characteristics of the system. Traditional filtration on a minimal level of expression and fold change will exclude these genes from initial examination. However, these genes may be considered at later stages of the analysis to clarify the biology behind gene expression changes.

To estimate the influence of fold change and expression level restrictions on the quality of analysis, we used the data described in the Materials and Methods as a presumably homogenous group of 20 samples split into two equal subgroups. One of the subgroups remained unchanged (control) and the other was subjected to the procedure of balanced changes described above (Table S2, S3, S4, S5 and S6). Data were Two-Step normalized and the same method of analysis – Associative analysis of differential gene expression [19] – was used for all comparisons.

**Figure 2:**
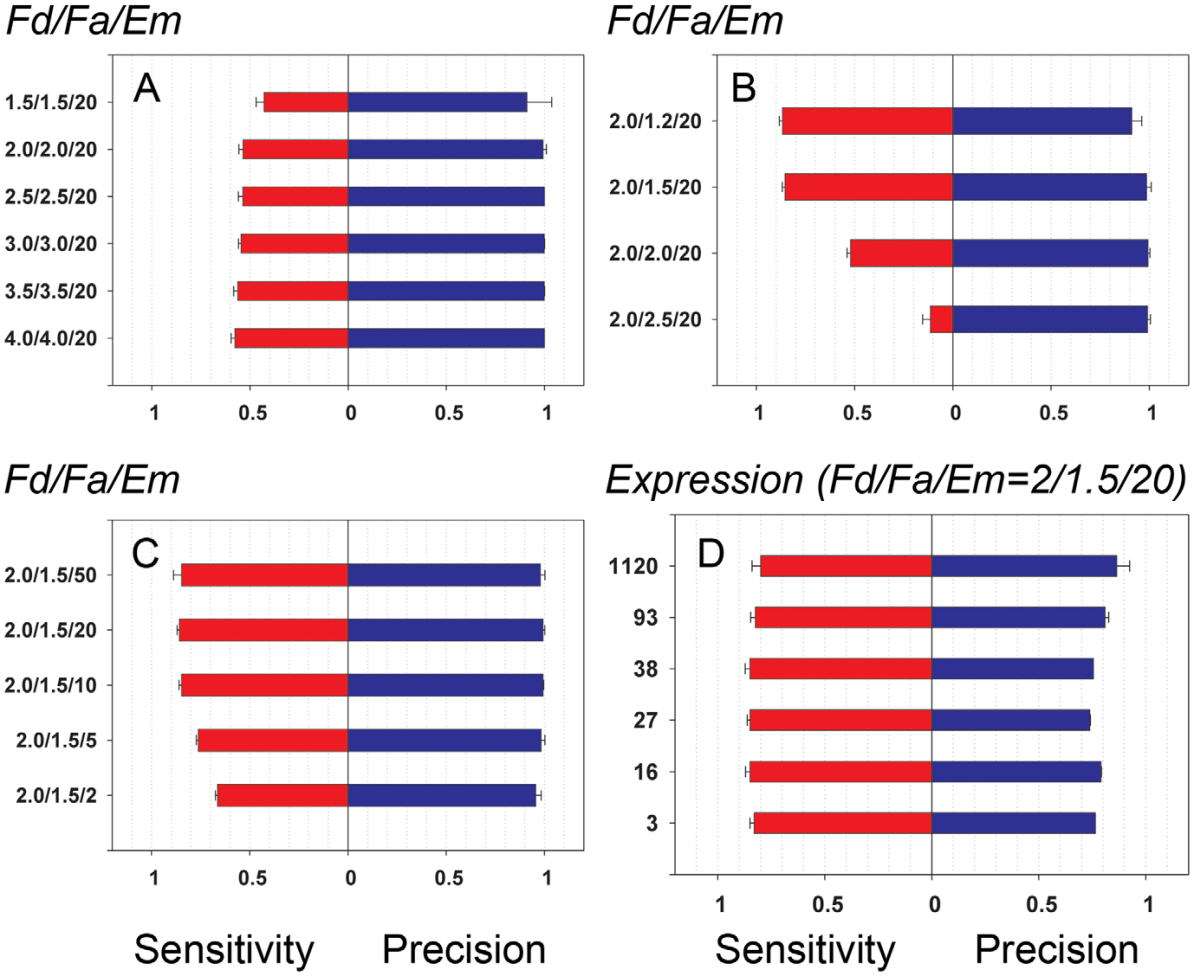
The influence of restrictions on the quality of differentially expressed genes detection (Fd - the level of introduced changes, analysis restrictions on the minimal level of expression – Em, and fold change Fa). All results were obtained by application of the Associative analysis (see Materials and Methods) to the analysis of data with controlled changes in expression of part of the genes. Sensitivity and Precision are shown with bars of red and blue color correspondingly. A–B) Dependence of the Sensitivity on the relative values of introduced changes (fold change Fd) and minimal fold change restriction for Associative analysis (Fa). Only fold change used in Associative analysis smaller than introduced fold change Fd yielded highest Sensitivity and Precision. C) Decrease of the Sensitivity with decrease of the minimal expression level to 2SD of the normal distribution of technical noise. D) Relative stability of the quality of estimations over the whole range of gene expression levels.

Figure 2 shows the effect of introduced fold change (Fd), restriction on the minimum fold change (Fa) and expression level (Em) on the Sensitivity and Precision of the differential expression analysis. Sensitivity is by far most affected by the choice of these parameters, whereas Precision remains relatively stable across all values. Deviation of these parameters from optimal values first leads to loss of positive selection. The procedure for selecting differentially expressed genes in the Associative analysis includes these restrictions. Violation of even one of them results in exclusion of that gene from the list of differentially expressed genes. This behavior explains why Sensitivity decreases as parameters of the analysis are changed. Naturally, it follows that under these conditions, it is more difficult to create conditions leading to increased levels of false positives. As a result, the Precision of the analysis method is more robust to variation in these restriction conditions.

Figure 2A demonstrates that the use of the same restrictions on the level of real fold change data and minimal fold restriction (Fd = Fa) in the Associative analysis leads to the loss of up to half of all differentially expressed genes. The pre-processing procedures (normalization, adjustment) are likely responsible for this loss because even a slight decrease in expression level can reduce the resulting fold change for a portion of genes below the established cut off (Fd resulting<Fa), thereby increasing the number of false negative selections. Figure 2B shows that, in the Associative analysis, as the restriction on fold change (Fa) approaches (or even exceeds) the real fold change (Fd), Sensitivity decreases significantly, as would be expected in this situation.

Figure 2C shows that, at constant Fd/Fa of 2.0/1.5, the Sensitivity of detection of differential expression drops as the restriction for the minimum expression level (Em) decreases. This behavior is due to excess of highly variable expression near the level of background noise. Figure 2D shows that the actual level of expression has relatively little effect on the performance of the Associative analysis.

In summary, the maximal level of Sensitivity does not exceeded 0.85 even under the best conditional situations. The controlled changes introduced into real expression data do not necessary make the changed expression really significantly different. The statistical tests for differential expression analysis are based on the difference in the expression level (Average) and its variation in replicates (SD). A proportion of genes with extremely high variation of the expression level is always present even in the very homogeneous group of samples. The introduction of even substantial difference for such genes does not cancel the fact of their extreme variability. Being significantly different in the level of expression (after controlled changes) such genes remain to be extremely variable, and as a result could not be selected as significantly different with any realistic differential analysis. The selection and exclusion from analysis of such genes (with methods presented in [19], [24]) were able to elevate the Sensitivity level in optimal variants in Figure 1 to the near 1 value (not shown) and should be considered in the course of any analysis.

### The influence of the number of replicates (Power analysis)

**Figure 3:**
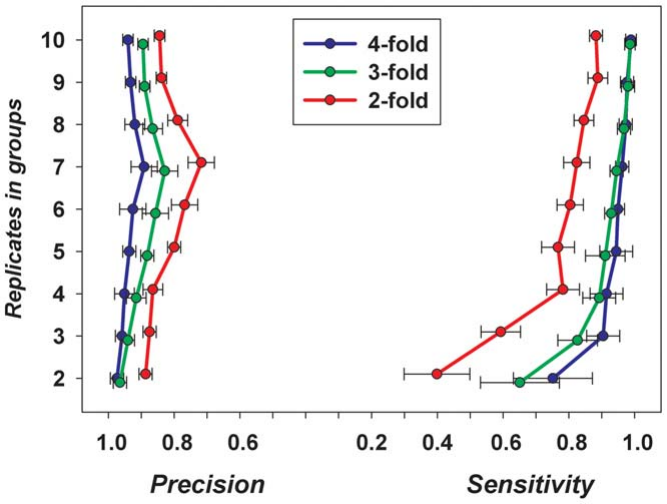
The influence of the number of replicates (Power analysis). Dependence of the quality of Associative analysis (Fa = 1.5, Em = 20) from the number of replicates is shown for different introduced fold changes Fd (X axis - Precision on the left side, and Y axis - Sensitivity on the right side). The results are obtained as an average of the analysis of three bootstrapped datasets and expressed as mean/SD.

The model presented here enables easy estimation of the dependence of analysis quality on the number of replicates. The results shown in Figure 3 demonstrate that in contrast to the typically observed decrease in Specificity with decreasing numbers of replicates (not shown) we observed no decrease in Precision as the number of replicates was reduced. At the same time, the Sensitivity of the analysis dropped significantly when the number of replicates in each group drops below 4–5. The information presented here enables us to estimate the number of replicates required for the desired performance in an experimental design. These estimations can be accurate and individually tailored for specific microarray technologies, sources of mRNA, quality of technological procedures, and other parameters. For example, in the analysis presented here, a minimum of 4 replicates are required to achieve about 80% Sensitivity for the detection of 2-fold differences. This finding is a consequence of the high quality of presented expression data and of the good performance of the analytical procedure (Two-step normalization & Associative analysis – see Materials and Methods). Larger fold changes can be identified with even higher accuracy with the same number of replicates.

The method presented in this study enables estimation of the number of replicates required by using information about real diversity in real preliminary data. All other methods of power analysis use averaged estimations of gene expression variation obtained in preliminary experiments.

### Comparison of data preprocessing (normalization, adjustment) and methods for differential gene expression analyses

The effect of noisy technical variation in gene expression level in the arrays can be minimized using normalization procedures. However, the choice of normalization method can have a substantial impact on the results of detection of differentially expressed genes. Our method enables assessment of different normalization techniques and their effects on the quality of the results.

**Figure 4:**
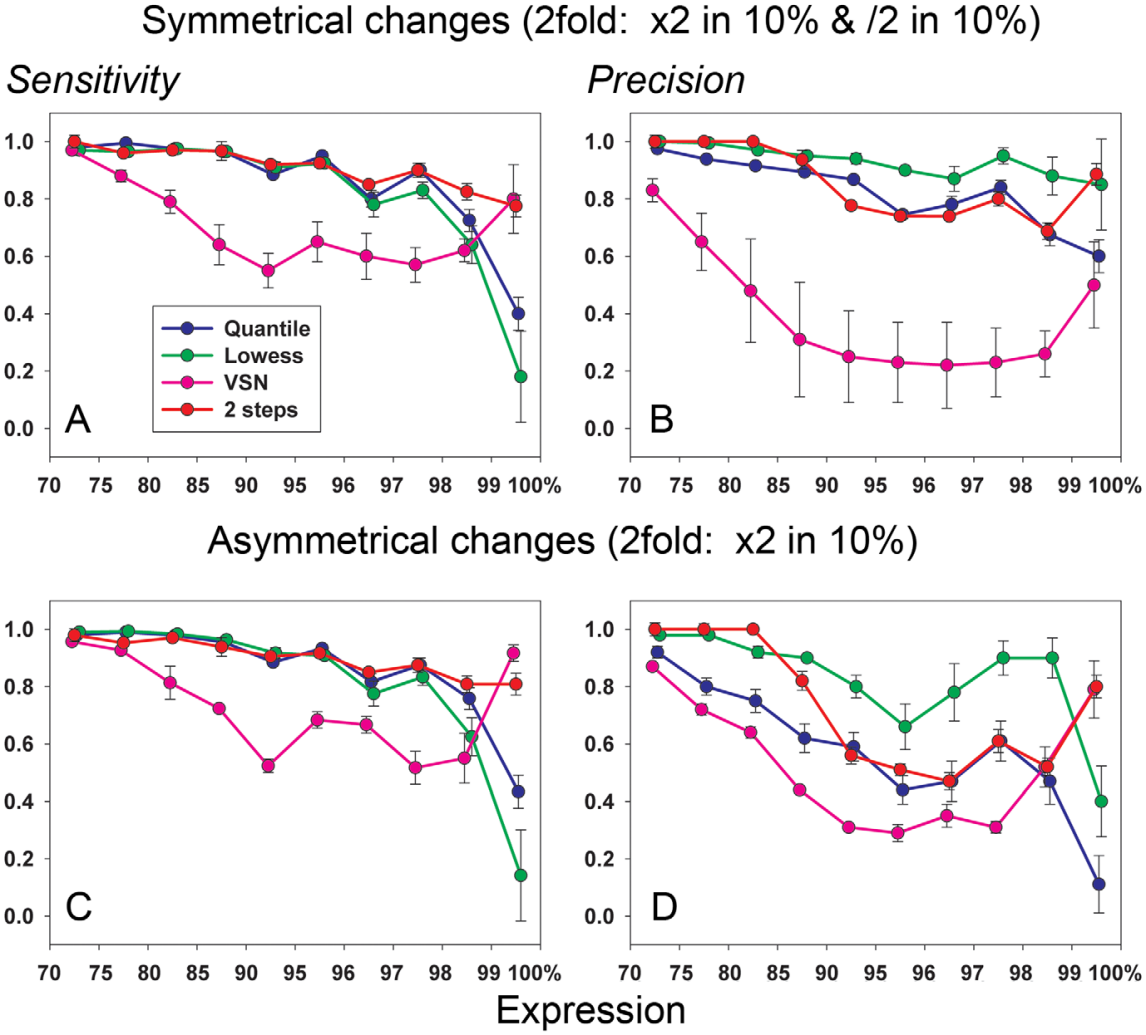
Comparison of normalization methods. The dataset was split into two equal subgroups, one of which remains unchanged and is used as a standard for comparison. The second subgroup is altered by introduced fold changes in the portion of gene expression. Four different normalizations were applied to the resulting data: Two-step normalization, Quantile, Lowess, and VSN. Associative analysis (see Materials and Methods) was used for the selection of differentially expressed genes with Fd/Fa/Em = 2/1.5/20 restrictions in all these cases. The procedure was repeated three times (every time with different arbitrary split of 20 samples data into 10&10 samples subgroups) and the averages and SD of the estimations are shown. A) Sensitivity and B) Precision (Y axes) for symmetrical changes in gene expression (2-fold increase in 10% and 2-fold decrease in another 10% of data). C) Sensitivity and D) Precision for asymmetric changes in gene expression (2-fold increases in 10% of gene expressions only). X axis shows the positions of the blocks of gene expression data used for the parameters estimations (in percentage along decreasingly sorted data, i.e. the 99–100 interval presents 1% of the genes with the highest expressions in the array).

Figure 4 compares the results of the Associative analysis applied to the data subjected to different methods of normalization. These comparisons (upper part of Figure 4) were performed using the 2-fold balanced changes applied to 20% of the data from the experimental subgroup (2-fold increase in 10% of the data and a simultaneous 2-fold decrease in 10% of the neighboring data in each 1000-gene block as shown in Figure 1, Supplemental Table S4). Examples of analysis of the data with 10% and 5% balanced changes (Supplemental Tables S2 and S3) are presented in Supplemental Figure S1. Both Quantile and Lowess normalizations resulted in loss of the Sensitivity on highest expression levels. VSN and 2-step normalizations have equally good Sensitivity in this area however for the rest of the expression levels VSN demonstrated lower levels and lower stability of Sensitivity compared with 2-step normalization. Little difference in Precision is seen for all three methods excluding VSN, which again shows drop in level and stability.

In case of asymmetrical changes there was practically complete loss of Sensitivity and Precision for Quantile and Lowess for genes with highest expression levels (Figure 4C, D). In fact there was severe degradation of the Precision in the analysis of the data normalized by all these procedures (Figure 4D).

**Figure 5:**
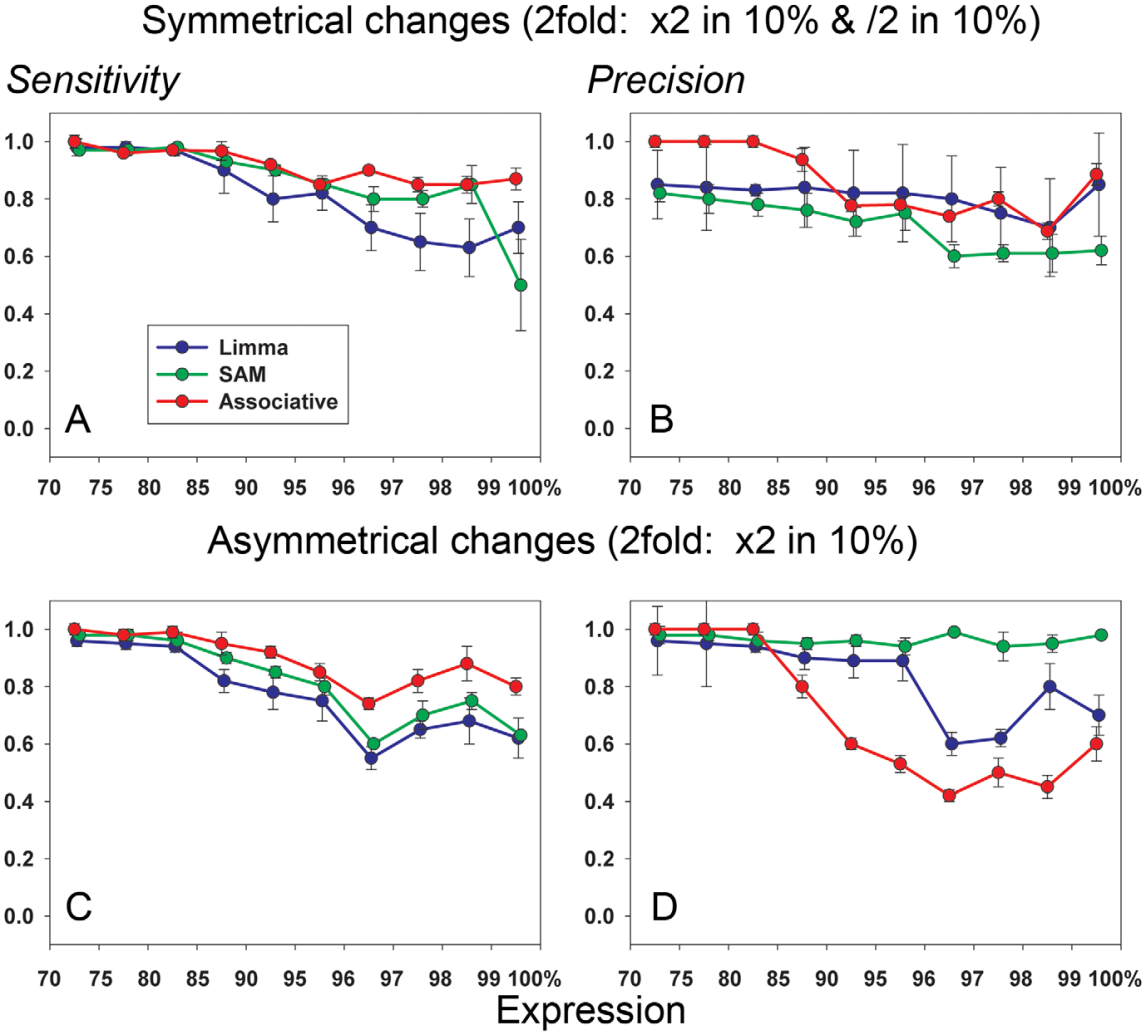
Comparison of different methods for gene expression analysis. Sensitivity/Precision of Limma, SAM and Associative analyses. The 2-step normalization procedure was used in all cases. The restrictions were Fd/Fa/Em = 2/1.5/20. A) Sensitivity and B) Precision for symmetrical changes in gene expression (2-fold increases and 2-fold decreases in gene expressions were equally presented). C) Sensitivity and D) Precision for asymmetric changes in gene expression (2-fold increases in 10% portion of gene expressions). Axes designation is the same as in Figure 4.

Several modified statistics have been proposed for analysis of the significance of differences in gene expression. Of these, SAM [25] is arguably the most popular. We compared SAM with two other procedures – Associative analysis [18], [19] and Limma [26]. These comparisons were performed using the 2-fold balanced changes applied the 20% of raw data (see above) with subsequent 2-step normalization (Materials and Methods). The results are shown in Figure 5 (Analyses for 10% and 5% are shown in the Supplemental Figure S2). All three methods demonstrate similar patterns of Sensitivity and Precision, however, Limma analysis produces much less reproducible results. The loss of reproducibility and decrease of Sensitivity were seen also in case 10% and 5% changes (Supplemental Figure S2A, C) for both SAM and Limma methods. Associative analysis demonstrates essential loss of Precision compared with other methods (Supplemental Figure S2B, D). The same loss of Precision was seen also in case of asymmetrical changes (2-fold increase in 10% data) – Figure 5D. We would like to note however, that all these positive conclusions about performance of SAM and Limma methods were obtained with use of 2-step normalization procedure, which demonstrated better performance in the most important area of highest gene expression (Figure 4 and Supplemental Figure S1). Published microarray data analyses usually used the combination of SAM and Limma methods with popular Quantile and Lowess normalizations. Poor performance of these normalizations at high gene expression area is able to deteriorate essentially the overall quality of such combinations in gene expression analyses (Figure 5).

## Discussion

Rapid development of microarray technology over the past decade has produced a number of methods for data pre-processing and selection that can be used to identify differentially expressed genes in microarray datasets. Importantly, different analytical methods frequently identify different lists of differentially expressed genes. Although numerous reviews have examined the details of a variety of different methods [5], [14], [25] only a few publications have addressed the issue of direct quantitative comparisons of different methods. Despite the fact that ROC-curves [1], [2], [3] can be used to characterize overall performance of different array platforms and software packages, practical use of this comparison is limited due to the lack of *a priori* knowledge of the number of true positive and true negative genes in a given dataset. Verification of the differences in gene expression using experimental techniques such as RT-PCR cannot solve this problem because this approach only verifies a subset of true positives but does not provide any information about the number of true and false positives in the entire dataset [25]. Several studies have analyzed simulated datasets containing a known number of truly regulated genes [5], [6], [7], [8], [9]. However, it is unclear whether these simulated datasets realistically reflect the complex structure of real microarray data. Progress has been achieved by using spike-in experiments that introduce controlled changes in hybridization/expression of a known portion of the genes in an array experiment. However, this technique remains expensive and restricted in its applicability. An essential drawback of the spike-in experiments involves the non-linearity of probe effects. Deviation from linearity near minimum detectable level and saturation level can misrepresent the results of the performance estimation [10]. This drawback is usually overcome by titration of the probes over a wide range of concentrations to obtain linear data for use in the comparative analysis. The necessity for titration makes this method expensive, lengthy and difficult for interpretation.

We have presented a methodology for quantitative estimation of the effects of pre-processing procedures and data analysis methods on the detection of true differentially expressed genes. The greatest strength of our strategy is the creation of test datasets through the introduction of controlled changes into real gene expression data. The methodology presented here is universal and can be applied to any existing microarray technology, any source of sample material and any experimental design. The use of real gene expression data that contains an unaltered microstructure (expression level profile, variation patterns, and even typical for the technology errors etc.) is a great advantage of this approach over similar attempts that have used simulated data or artificial models based on the use of simplified/averaged statistical characteristics from real experiments. Introduction of controlled changes into the expression dataset enables us to address many problems associated with pre-processing and analysis of microarray data, including the quality dependence of the analysis from the threshold of minimal gene expression, the influence of fold change restrictions, and other factors. Further, the introduction of controlled changes completely eliminates the problem of non-linearity of probe effects because the changes are introduced into the final expression levels as if they were measured in the linear region of the dose/response dependence curve. We believe that the use our comparative approach will improve the robustness of microarray-based experiments.

Homogeneous real gene expression data from any experiment can be used to create a transformed dataset with controlled balanced changes introduced into a portion of the genes measured in the array. Similar microarray data matching the manufacturer, organism, tissue/cells can almost always be found among >400,000 microarray samples stored on Gene Expression Omnibus (GEO, [16]), if such data were not included in the initial experimental design.

**Figure 6:**
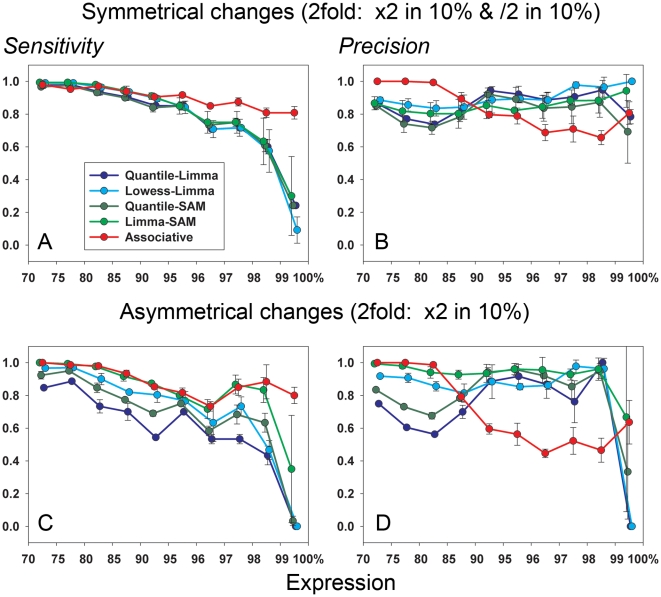
Comparison of different methods for gene expression analysis in conjunction with normalization procedures usually used with these methods. The results are shown for symmetric (A, B) and asymmetric (C, D) changes in gene expressions for combinations Quantile&Limma, Lowess&Limma, Quantile&SAM, Lowess&SAM, and 2-step normalization&Associative analysis. All details of experiments and designations are as in Figure 5.

Homogeneity of the initial data set will influence estimations of the performance of the analyses. Estimation of true and false selections are based on the proposition that after arbitrarily chosen split the resulting subgroups have only changes in gene expression produced mainly by the controlled modifications in one of the subgroups. In practice, however, we have the presence of various types of outliers even within most homogeneous biological data. The approach presented here is especially sensitive to the presence of genes having extremely variable expressions [27], which could not be presented equally after arbitrary split of the group into two subgroups for subsequent controlled changes and analysis. The best strategy to estimate and minimize the influence of such genes is to repeat the procedure several times with random permutation of the order of samples within the group before each split. The results (Sensitivity/Precision) will be presented in form of means+/− SD as shown in Figures 2, 4, 5 and 6. The “outliers” in our data did not interfere essentially with our tests, as very small variation in the Sensitivity/Precision estimations was seen in many cases (see for example 2–step normalization in conjunction with Associative analysis – Figures 4, 5). Still, the “outliers” can make more significant influence in some practical cases, especially associated with the analysis of quite heterogeneous clinical data. However, even in such cases it is possible to compare different methods of analysis as they will be still in the same equal conditions. The presence of “outliers” is a reality of practical analysis that usually is not estimated and ignored. The use of the multiple arbitrary splits and analysis of resulting differences in gene expressions between “equal” subgroups (before introduced changes) will help to observe real non-homogeneity and estimate its contribution into all subsequent results.

To demonstrate the potential of our proposed methodology we performed quantitative comparisons of the efficiencies of different normalization and analysis methods. The presented results enable to compare the performance of all these methods quantitatively in the wide ranges of gene expression levels and estimate stability of the characteristics of the performance (Sensitivity and Precision). This analysis is based on the use of preliminary data. However, if the user is unable to run several microarray experiments to estimate the robustness of specific pre-processing and statistical analysis methods, real data that mimic the experimental conditions can be freely obtained from public microarray data repositories (GEO [16], ArrayExpress [28]).

The question ‘how many replicates is enough?’ is complicated by many potentially confounding factors such as the type of array equipment, laboratory technique, and, most importantly, the quality of the samples. Cost often represents a significant restraint and it is important to know which fold changes can be detected reliably for a given number of replicates. Our analytical approach can be used to objectively estimate the number of replicates of a microarray experiments required to reach any desired quality of analysis and can be completely adjusted to the technological platform and experimental design.

In summary, we present an accurate and universal procedure for quantitative and qualitative estimation of the methods of microarray data analysis. Our approach has the potential for broad applicability to different types of arrays, including those with asymmetric distributions of up/down-regulated genes. All programs used for our analysis were written in MATLAB and are available upon request. The microarray data containing balanced 2-fold differences in the levels of expression of 20% of the genes measured are provided as Supplemental material (Table S1) and may be used by readers for quality analysis estimation of their own analytical methods. The main result of the presented here analyses were reproduced without essential differences with smaller proportion of the controlled changes (5% and 10%, Supplemental material).

## Supporting Information

Figure S1 Comparison of normalization methods. Two-step normalization vs. Quantile, Lowess, and VSN normalizations. All designations are as in Figure 4. Associative analysis was used for the selection of differentially expressed genes with Fd/Fa/Em = 2/1.5/20 restrictions in all cases. A) Sensitivity and B) Precision (Y axis) for 2-fold symmetric changes of 10% gene expressions. C) Sensitivity and D) Precision for symmetric changes in 5% gene expressions.
(0.73 MB TIF)

Figure S2 Comparison of different methods for gene expression analysis. Limma, SAM and Associative analysis performance compared in terms of Sensitivity/Precision. 2-step normalization procedure was used in all cases. The restrictions were Fd/Fa/Em = 2/1.5/20 as before. A) Sensitivity and B) Precision for symmetric changes in 10% of gene expression; C) and D) - the same for 5% changes.
(0.58 MB TIF)

Table S1 Raw data (before normalization) without introduced changes. In this research we used the group of arrays created from 20 samples from Epstein-Barr Virus (EBV)-transformed B cells collected from normal healthy donors (Illumina Whole Genome, Human Ref-8 V2.0 arrays containing over 20,000 genes). This presumably homogenous group was split into two equal subgroups. One of the subgroups (columns Q-Z) was used as a control, whereas the artificial changes in gene expressions were introduced in another subgroup (experimental - G-P columns). All data were sorted by the average expression in experimental group.
(6.30 MB XLSX)

Table S2 Data with introduced symmetrical 2-fold differences in 5% of the expression in one half of samples. Column AA (Modifications) shows the changed gene expression: 1 - means 2-fold increase of expression, and 2- 2-fold decrease, zero means unmodified data. Data with increase/decrease of expression are highlighted red/blue, respectively.
(6.45 MB XLSX)

Table S3 Data with introduced symmetrical 2-fold differences in 10% of the expression in one half of samples. Column AA (Modifications) shows the changed gene expression: 1 - means 2-fold increase of expression, and 2- 2-fold decrease, zero means unmodified data. Data with increase/decrease of expression are highlighted red/blue, respectively.
(6.46 MB XLSX)

Table S4 Data with introduced symmetrical 2-fold differences in 20% of the expression in one half of samples. Column AA (Modifications) shows the changed gene expression: 1 - means 2-fold increase of expression, and 2- 2-fold decrease, zero means unmodified data. Data with increase/decrease of expression are highlighted red/blue, respectively.
(6.41 MB XLSX)

Table S5 Data with introduced asymmetrical 2-fold increase only in 10% of the expression in one half of samples. Column AA (Modifications) shows the changed gene expression: 1 - means 2-fold increase of expression, zero means unmodified data. Data with increase of expression are highlighted red.
(6.45 MB XLSX)

Table S6 The raw data (before normalization) with introduced asymmetrical 4-fold increase 10% of the expression and 2-fold decrease in another 10% of the expression in one half of the data. Column AA (Modifications) shows the changed gene expression: 1 - means 4-fold increase of expression, 2 - means 2-fold decrease of expression, zero means unmodified data. Data with increase/decrease of expression are highlighted red/blue, respectively.
(6.30 MB XLSX)
