# Supplementary figures and images for: A Comprehensive and Universal Method for Assessing the Performance of Differential Gene Expression Analyses
Source: PLoS One. 2010 Sep 9;5(9):e12657. doi: 10.1371/journal.pone.0012657 (PMC2936572; doi:10.1371/journal.pone.0012657)

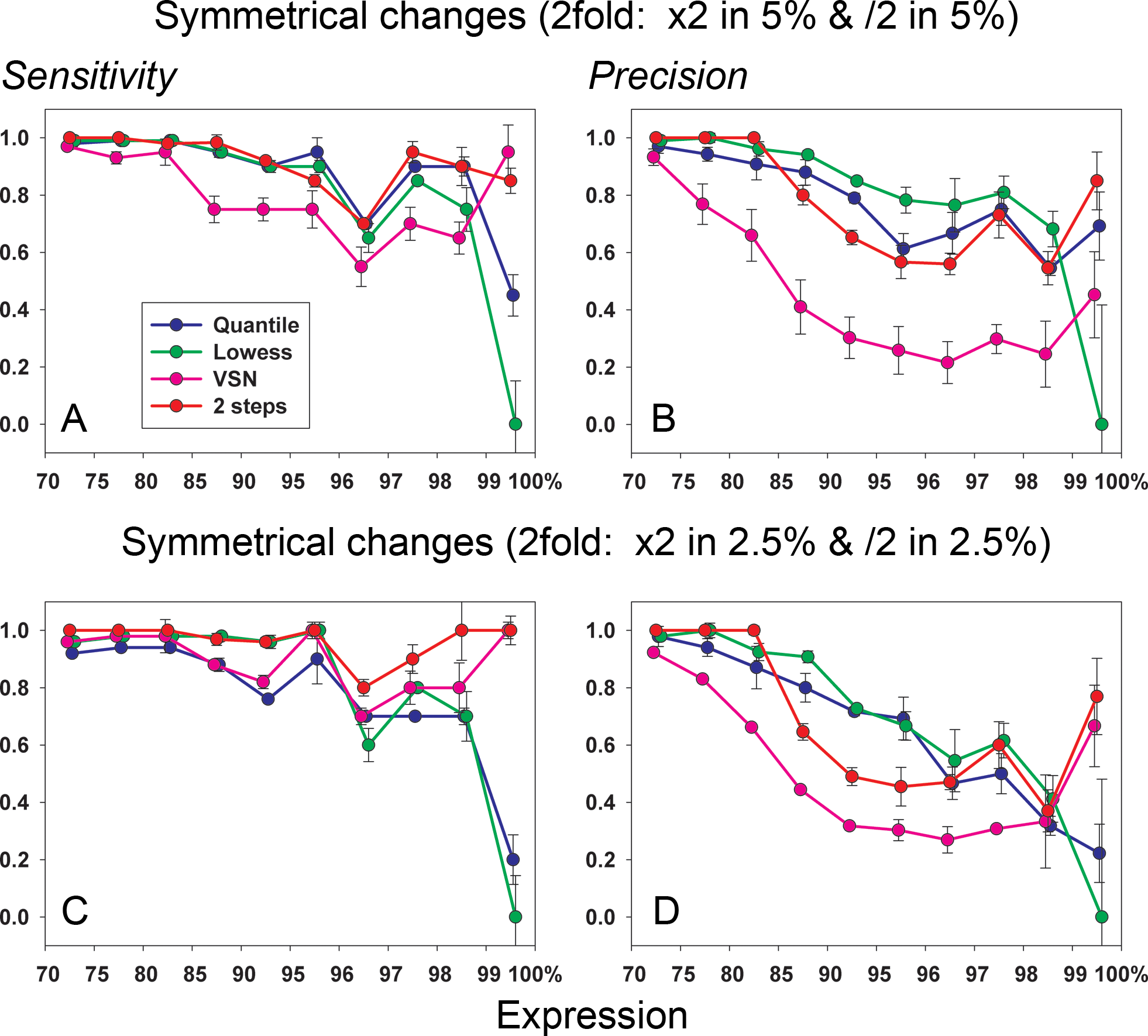

Supplement: Figure S1 — Comparison of normalization methods. Two-step normalization vs. Quantile, Lowess, and VSN normalizations. All designations are as in Figure 4. Associative analysis was used for the selection of differentially expressed genes with Fd/Fa/Em = 2/1.5/20 restrictions in all cases. A) Sensitivity and B) Precision (Y axis) for 2-fold symmetric changes of 10% gene expressions. C) Sensitivity and D) Precision for symmetric changes in 5% gene expressions. (0.73 MB TIF) [file pone.0012657.s001.tif]

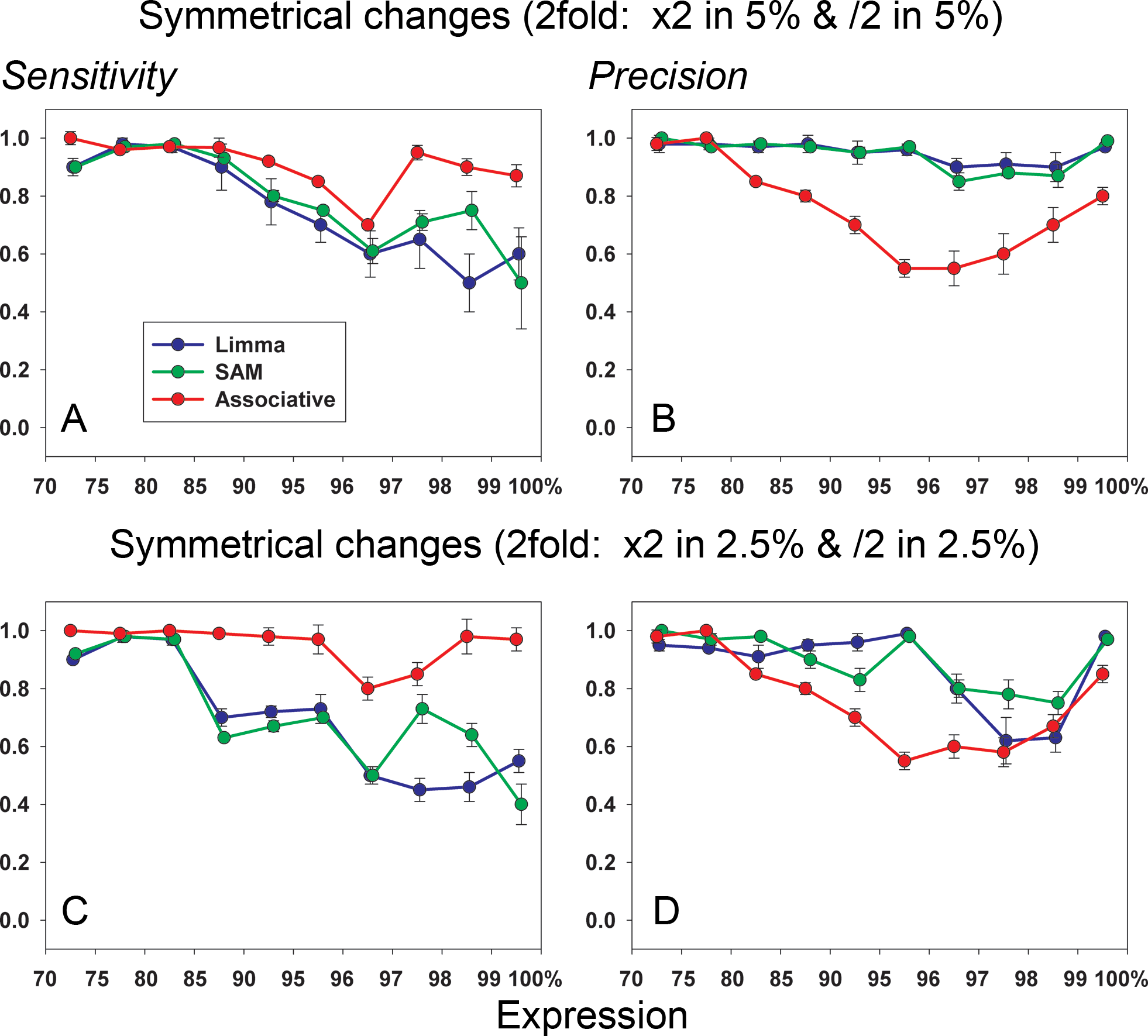

Supplement: Figure S2 — Comparison of different methods for gene expression analysis. Limma, SAM and Associative analysis performance compared in terms of Sensitivity/Precision. 2-step normalization procedure was used in all cases. The restrictions were Fd/Fa/Em = 2/1.5/20 as before. A) Sensitivity and B) Precision for symmetric changes in 10% of gene expression; C) and D) - the same for 5% changes. (0.58 MB TIF) [file pone.0012657.s002.tif]
